# Numerical Modeling and Experimental Validation by Calorimetric Detection of Energetic Materials Using Thermal Bimorph Microcantilever Array: A Case Study on Sensing Vapors of Volatile Organic Compounds (VOCs)

**DOI:** 10.3390/s150921785

**Published:** 2015-08-31

**Authors:** Seok-Won Kang, Joe Fragala, Debjyoti Banerjee

**Affiliations:** 1 Korea Railroad Research Institute, 176 Cheoldo bangmulgwan-ro, Uiwang, Gyeonggi-do 16105, Korea; 2 NanoINK, Inc., 215 E Hacienda Ave., Campbell, CA 95008, USA; E-Mail: joefrag9@outlook.com; 3 Department of Mechanical Engineering, Texas A&M University, College Station, TX 77843-3123, USA; E-Mail: dbanerjee@tamu.edu

**Keywords:** MEMS, NEMS, auto-ignition temperature, catalytic combustion, optical lever, CFD, CHT, computational analyses, experimental validation

## Abstract

Bi-layer (Au-Si_3_N_4_) microcantilevers fabricated in an array were used to detect vapors of energetic materials such as explosives under ambient conditions. The changes in the bending response of each thermal bimorph (*i.e.*, microcantilever) with changes in actuation currents were experimentally monitored by measuring the angle of the reflected ray from a laser source used to illuminate the gold nanocoating on the surface of silicon nitride microcantilevers in the absence and presence of a designated combustible species. Experiments were performed to determine the signature response of this nano-calorimeter platform for each explosive material considered for this study. Numerical modeling was performed to predict the bending response of the microcantilevers for various explosive materials, species concentrations, and actuation currents. The experimental validation of the numerical predictions demonstrated that in the presence of different explosive or combustible materials, the microcantilevers exhibited unique trends in their bending responses with increasing values of the actuation current.

## 1. Introduction

Miniaturized sensors have enormous potential for use in detecting small quantities (*i.e.*, in the pico/femtoliter range) and small concentrations (*i.e.*, in the pico/femtomolar range) of analytes, typically by the transduction of physical or chemical property changes of specific materials into very small ranges of motion (*i.e.*, in the nanometer range). For example, microcantilever-based sensing platforms are considered attractive for their one-dimensional response and their ease of analysis [1]. Microcantilevers have been widely studied as chemical and biological sensors [2,3,4,5,6,7,8,9,10,11] in which static bending induced by differential surface stress or changes in resonant frequency upon mass uptake is monitored [4,5,6,7]. A large body of literature exists on various types of microcantilever or N/MEMS (nano sensors used for chemical and explosives sensing) [8,9,10,11]. In addition, it has been reported that the sensitivity and selectivity of microcantilevers to specific explosive vapors can be enhanced through surface functionalization using nano-structured materials [12,13,14]. Microcantilever sensors are attractive for their fast thermal actuation characteristics (e.g., thermo-mechanical response times of ~1 ms). Typically, these microcantilever sensors utilize a polymer film for non-specific adsorption of the analytes of interest [7,11]. The adsorbed species induce surface stress on the microcantilever, causing a fast bending response (typically with a time constant in the range of 1 ms to 1 s). The fast sensor response for detection is handicapped by the long time required for desorption and return to the baseline response of the sensor (typically 5–6 h). In addition, non-specific adsorption and change under ambient conditions (e.g., temperature and humidity) can cause a creep in the sensor response, which can be rectified in laboratory testing but can be very difficult to alleviate under typical operational conditions.

**Figure 1:**
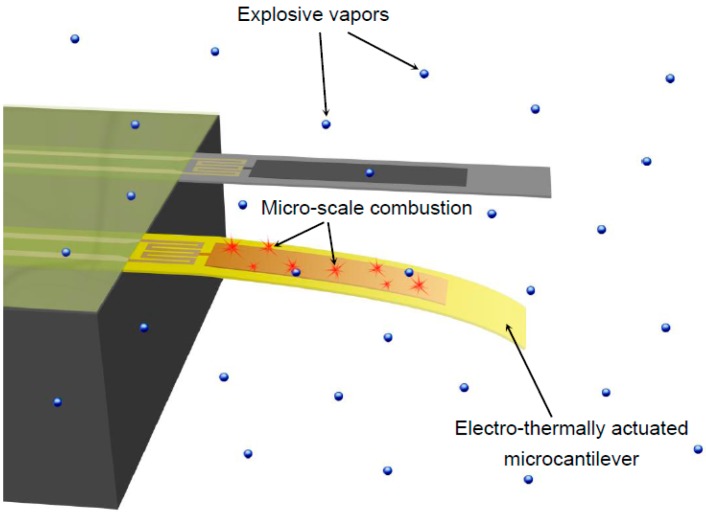
Schematic representation of the sensing mechanism proposed in this study.

Hence, in this study, an alternative detection scheme was used in which thermal bimorph microcantilevers were used effectively as nano-calorimeters for sensing the heat of reaction due to catalytic combustion of explosive vapors adsorbed on the sensor surface (as shown in Figure 1). In general, the exothermic reactions due to combustion of the species in the vapor phase (emanating from the energetic materials that are adsorbed on the surface of a microcantilever) cause changes in surface stress profiles in the bi-layer microcantilever (due to adsorption, catalytic oxidation, and temperature gradients caused by various modes of heat transfer). These changes result in thermo-mechanical (bending) responses of the microcantilever sensor. The objective of this study was to garner fundamental insights into the effects of the various transport mechanisms that are coupled with the reaction kinetics of the various species (generated from the intermediate chemical reactions during the catalytic oxidation of the explosive materials) on the thermo-mechanical response of the microcantilever sensor platform. The fundamental insights garnered in this study can be used to improve the detection schemes for various energetic materials (e.g., explosives, combustibles, propellants, *etc.*) using this nano-calorimeter platform. The results of this study can also contribute to the optimization of the device architectures of thermal bimorphs and microcantilever sensors.

To initiate the catalytic oxidation of the adsorbed vapor species, the microcantilever surface is heated electrically (*i.e.*, ohmic heating or Joule heating). This is accomplished by a gold micro-heater that is patterned and fabricated *in situ* at the base of each silicon nitride microcantilever with a gold nanocoating. A mixture of air and chemical species in the vapor phase (emanating from the explosive materials) is transported by diffusion onto the microcantilever surface and is progressively heated by the temperature gradients on the microcantilever surface, in the axial and transverse directions as well as on both the top and bottom surfaces in the direction normal to the surface. The temperature gradients are caused by various modes of heat transfer that are induced by Joule heating in the micro-heater and by the exothermic reaction on the catalyst surface. Eventually, if the surface temperature of the microcantilever exceeds the auto-ignition temperature of the chemical species in the vapor phase, combustion reactions are also initiated in the gas phase. Hence, the thermal response of microcantilevers caused by chemical reactions is determined by the competing effects of chemical kinetics and thermal diffusion.

In this study, experimental measurements were performed to validate predictions obtained from a numerical model used to simulate the response of the nano-calorimeter platform for detecting explosive vapors. Volatile organic compounds (VOCs) (e.g., acetone and 2-propanol) were selected for use in this study because of the availability of chemical reaction modeling parameters that are amenable to computational fluid dynamics (CFD) simulations. The catalytic oxidation reactions were simulated using a heterogeneous reaction model. The model was applied to a thin film of heated metal (*i.e.*, Au) by coupling the species transport models for diffusion with gaseous (*i.e.*, homogeneous) reactions. Because the Au catalysis enables ultra-lean oxidation, detection of species in the vapor phase is possible at very low concentrations (*i.e.*, the picomolar to femtomolar range) [10]. Catalytic oxidation occurs because of lowering of the activation energy (or temperature) in comparison to that of the homogeneous case [15]. During the commencement of heterogeneous catalysis, the initial products from the intermediate chemical reactions diffuse away from the microcantilever surface and are proportional to the initial concentration gradient [16]. The deflection (bending response) of the microcantilevers was measured experimentally using an optical lever, by tracking the light spot reflected from the microcantilever surface that is illuminated by a laser source.

## 2. Numerical Modeling and Simulation

The flow regime is assumed to be steady, incompressible, and laminar. The gas mixture is treated as an ideal gas. The governing equations used in this study were the continuity (or conservation of mass) equation, the equation of motion (or momentum conservation), the energy balance (or thermal energy) equation, and the mass balance (or material balances, or species) equation:

(1) $$\nabla •\vec{v}=0$$

(2) $$\left(\vec{v}•\nabla \right)\vec{v}=-\frac{1}{\rho }\nabla p+\frac{\mu }{\rho }\nabla^{2}\vec{v}$$

(3) $$\nabla •\left(\rho \vec{v}Y_{i}\right)=\frac{\partial }{\partial x_{j}}\left(\rho D_{i,m}\nabla Y_{i}\right)+R_{i};\;i=1,2,\ldots ,N_{s}$$

(4) $$\nabla •\left(\rho H\vec{v}\right)=-\nabla •q+\mu {\text{Φ}}_{v}$$

where $\vec{v}$, *p*, *μ*, *ρ*, *Y_i_*, *R_i_*, *N_s_*, *H*, *q*, and *Φ_v_* represent the velocity of the components (m/s), the pressure (Pa), the viscosity (Pa·s), the density (kg/m^3^), the mass fraction of the species, the rate of reaction of species (mol/m^3^-s), the number of species, the total enthalpy (J/mol), the heat flux due to conduction and species diffusion (W/m^3^), and the viscous dissipation (N/m^2^-s), respectively.

### 2.1. Analytical Model

A one-dimensional (1-D) model was formulated to calibrate the governing equations used in the CFD code and to understand the thermal response of the sensor when the gas concentration (e.g., the mole fraction or mass fraction) is initially specified as a constant. For situations involving “fast” reaction kinetics (*i.e.*, diffusion-limited reactions) the concentration of an explosive species over the surface can be obtained by solving the 1-D species diffusion equation, as shown below:

(5a) $$D_{i,air}\frac{d^{2}C_{i}}{dx^{2}}-k_{v}C_{i}=0$$

where

(5b) $$D_{i,air}=\frac{1.013\times 10^{-2}T^{1.75}{\left[\frac{M_{i}+M_{air}}{M_{i}M_{air}}\right]}^{0.5}}{p{\left[V_{i}^{1/3}+V_{air}^{1/3}\right]}^{2}}$$

where *D_i,air_* is the effective binary mass diffusion coefficient (m^2^/s) for the explosive species that is diffusing in a mixture with air, *p* is the pressure (Pa), *T* is the temperature (K), *M* is the molecular weight (kg/kmol), *V* are the diffusional volumes (m^3^) [17], *k_v_* is the chemical reaction constant (s^−1^), and *C_i_* is the molar concentration (kmol/m^3^). The chemical reaction constant, *k_v_*, is modeled based on first-order homogeneous reactive flow and is proportional to the volumetric concentrations (kmol/m^3^) of each species. This is conventionally approximated by the Arrhenius model, as expressed below:

(6) $$k=AT^{\beta }\exp\left(-\frac{E}{RT}\right)$$

where *A* is the pre-exponential factor (s^−1^), *β* is the temperature exponent, *E* is the activation energy (J/kmol), and *R* is the universal gas constant (J/kmol-K). The chemical reaction model for surface reactions (heterogeneous reactions) was developed using a procedure very similar to that used for the gas-phase reactions (homogeneous reactions). The boundary conditions applicable to this system are as follows:

(7) $$C_{i}=C_{i,0},\text{ at }x=0\;(\mathrm{in}\;\mathrm{gas})\;D_{i,air}\frac{dC_{i}}{dx}=-k_{s}C_{i},\text{ at }x=L\;(\mathrm{in}\text{ catalyst})$$

where *C_i,0_* is the initial concentration of explosives in the control volume. If evaporation occurs in a state of dynamic equilibrium (*i.e.*, assuming the explosive vapor saturates the ambient air), the initial concentration is obtained from the partial pressure of each species (*i.e.*, using Dalton’s law of partial pressures).

### 2.2. Chemical Kinetics

The complete oxidation reaction of VOCs is highly exothermic. The global oxidation models used in the numerical simulations for the different gases considered in this study are summarized in Table 1. The gas-phase reaction scheme was adapted from information in various reports in the literature. The model was based on first-order homogeneous reactive flow, where the rate of reaction is proportional to the volumetric concentrations of each constitutive species. The gas-phase reaction of VOCs leads to the formation of CO_2_ or H_2_O for complete oxidation conditions. In addition, at low temperatures in particular, VOCs oxidation causes the formation of intermediate products (*i.e.*, for acetone, it leads to the formation of CO, while for 2-propanol, it leads to the formation of CO, C_3_H_6_, or C_3_H_6_O). The multiple-step combustion model shown in Table 2 provides a more realistic description of the chemical kinetics than the “global” one-step reaction model.

**Table 1:** Global one-step reaction models, saturation pressure, and mole fraction in air of acetone and 2-propanol.

Gases	Combustion Model	Heat of Combustion	*P_i_*^sat^ (mmHg)	*Y_i_*
Acetone (CH_3_)_2_CO	${\left({\text{CH}}_{3}\right)}_{2}\text{CO }+{\text{ 4O}}_{2}\to 3{\text{CO}}_{2}+3{\text{H}}_{2}\text{O}$	–1761 kJ/mol (–303.2 × 10^5^ J/kg)	184.950 A = 7.2316 B = 1277.03 C = 237.23	0.243
2-Propanol C_3_H_7_OH	$2{\text{C}}_{3}{\text{H}}_{7}\text{OH}+9{\text{O}}_{2}\to 6{\text{CO}}_{2}+8{\text{H}}_{2}\text{O}$	–1907 kJ/mol (–317.3 × 10^5^ J/kg)	33.158 A = 8.1182 B = 1580.92 C = 219.62	0.044

It has been demonstrated that the activation energy of VOCs for catalytic oxidation can be expressed as a function of the molecular weight (MW) [18]. However, this relationship does not account for catalyst-dependent properties, such as the high selectivity of thin Au film in catalytic oxidation [19]. In general, the selectivity decreases with increasing temperature; hence, the complete oxidation pathway becomes dominant at higher temperatures [20]. In this study, complete oxidation on the catalyst surface was assumed to occur. The catalytic conversion of 2-propanol to acetone is usually initiated in the lower temperature regions. Deep oxidation (complete oxidation) typically occurs as the temperature increases. Thus, the reactions for the partial oxidation to acetone, as well as the complete oxidation of acetone and 2-propanol were implemented in the simulations involving 2-propanol.

**Table 2:** Chemical kinetic parameters for (multi-step) gas-phase reaction of acetone and 2-propanol (*P* = 1 atm).

Reaction	*A_r_*	*E_r_*
${\text{C}}_{3}{\text{H}}_{6}\text{O}+2.5{\text{O}}_{2}\to 3\text{CO}+3{\text{H}}_{2}\text{O}$	4.0 × 10^14^	2.09 × 10^8^
${\text{H}}_{2}+0.5{\text{O}}_{2}\to {\text{H}}_{2}\text{O}$	7.0 × 10^13^	8.79 × 10^7^
$\text{CO + 0.5}{\text{O}}_{2}\to {\text{CO}}_{2}$	8.5 × 10^12^	8.79 × 10^7^
$\text{CO}+{\text{H}}_{2}\text{O}\to {\text{O}}_{2}+{\text{H}}_{2}$	1.0 × 10^12^	1.74 × 10^8^
	3.1 × 10^13^	2.05 × 10^8^
${\text{C}}_{3}{\text{H}}_{7}\text{OH}\to {\text{C}}_{3}{\text{H}}_{6}+{\text{H}}_{2}\text{O}$	1.26 × 10^13^	1.06 × 10^8^
${\text{C}}_{3}{\text{H}}_{7}\text{OH + 0.5}{\text{O}}_{2}\to {\text{C}}_{3}{\text{H}}_{6}\text{O}+{\text{H}}_{2}\text{O}$	1.0 × 10^14^	1.05 × 10^8^
${\text{C}}_{3}{\text{H}}_{6}\text{+ 4.5}{\text{O}}_{2}\to 3{\text{CO}}_{2}+3{\text{H}}_{2}\text{O}$	6.75 × 10^9^	1.256 × 10^8^

The parameters for the activation energy for each metal catalyst are different [20], and the parameters for the reaction kinetics involving the complete catalytic oxidation of acetone over pure Au are not available in the literature. In this study, the activation energy for complete oxidation of acetone over Au was assumed to be 78.65 kJ/mol, based on a correlation [18] between the activation energy and molecular weight of two different VOCs derived from experimental data for methanol (CH_3_OH: 32.04 g/mol and 87 kJ/mol) [20] and propene (C_3_H_6_: 42.08 g/mol and 83.7 kJ/mol) [21]. The complete or partial oxidation of 2-propanol over metal catalysts has been reported in a previous study [19]. In this model, at a low temperature (*i.e.*, 393 K for Au/iron oxide catalysts) the catalytic oxidation of 2-propanol begins with a dehydration reaction that yields propene, as well as the formation of the dehydrogenated product (acetone) [22]. The complete oxidation from acetone (or propene) to carbon dioxide (CO_2_) and water vapor (H_2_O) occurs at a high temperature (*i.e.*, 553 K for Au/iron oxide catalysts) [23]. However, because of the high selectivity to acetone of 2-propanol in the pure gold catalytic oxidation process [19], the deep oxidation processes of 2-propanol to carbon dioxide and via acetone were considered together. The activation energy for the partial oxidation of 2-propanol was obtained from the literature as 1.6 ± 1.9 kJ/mol [19]. The surface reaction of these VOCs and their chemical kinetics parameters are listed in Table 3. The parameters listed in Table 3 were used in the models involving oxidation of 2-propanol.

Catalysts are substances that accelerate reactions without being consumed. Catalysts change reaction rates by offering different paths or mechanisms for the reaction. Catalysts change the speed of reactions. However, they do not affect the equilibrium. As shown in Equation (6), the reaction rate is a function of the pre-exponential factor and the activation energy. The pre-exponential factor, *A_s_*, is usually determined from experimental measurements. Three different approaches (*i.e.*, collision theory, collision theory combined with empirical data, and activated complex theory) were used for the determination of *A_s_* [18]. Because the values obtained from the “activated complex theory” were in good agreement with the experimental results [18], this approach was selected for use in this study. The values of *A_s_* for partial oxidation of 2-propanol (*i.e.*, conversion to acetone, as listed in Table 3) were selected from experimental measurements.

**Table 3:** Chemical kinetic parameters for heterogeneous surface reaction of acetone and 2-propanol.

Reaction	*A_r_*	*E_r_*
${\text{C}}_{3}{\text{H}}_{6}\text{O}+4{\text{O}}_{2}\to 3{\text{CO}}_{2}+3{\text{H}}_{2}\text{O}$	4.19 × 10^10^	7.865 × 10^7^
${\text{C}}_{3}{\text{H}}_{7}\text{OH}+4.5{\text{O}}_{2}\to 3{\text{CO}}_{2}+4{\text{H}}_{2}\text{O}$	3.54 × 10^10^	7.802 × 10^7^
${\text{C}}_{3}{\text{H}}_{7}\text{OH}+0.5{\text{O}}_{2}\to {\text{C}}_{3}{\text{H}}_{6}\text{O}+{\text{H}}_{2}\text{O}$	4.40 × 10^1^	1.6 × 10^6^

### 2.3. Numerical Methodology

**Figure 2:**
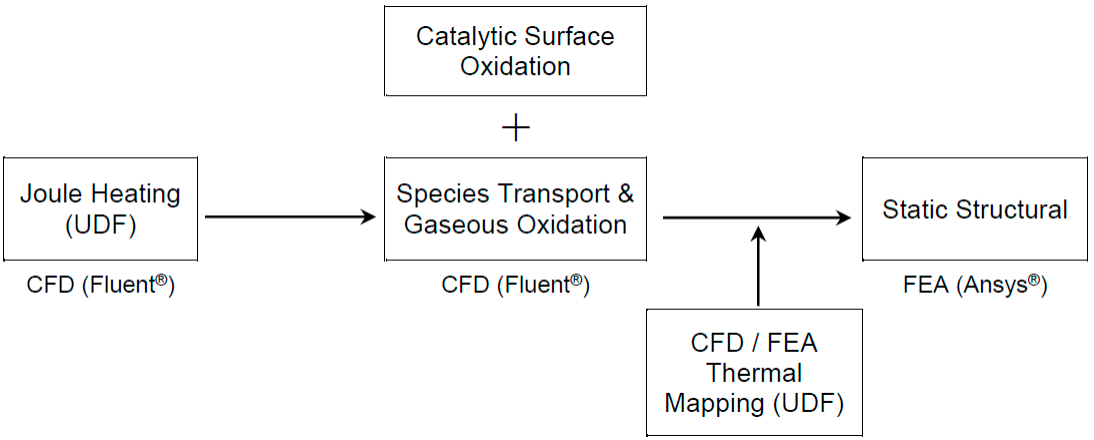
Schematic of a complete model of an electro-thermally actuated microcantilever.

A simulation model was developed and used to perform a parametric study of the coupled electro-thermo-mechanical models of the microcantilever platform used in this study. Correct estimation of the temperature profile of the microcantilevers is a key factor in characterizing chemo-mechanical sensing of explosives. The numerical model developed in this study can be used to predict the mechanical deflection of a bimorph structure in response to a change in the temperature distribution of the structure due to a chemical reaction. The procedure shown schematically in Figure 2 was implemented by coupling of a CFD tool (Fluent^®^) with a finite element analysis (FEA) tool (Ansys^®^). The final deflection values were obtained from a structural dynamics simulation (performed using Ansys^®^) in which the temperature data results obtained from the chemical reaction model (obtained using Fluent^®^) were mapped onto each finite element (FE) node.

#### 2.3.1. Computational Fluid Dynamics

The main assumptions underlying the numerical model are as follows: (i) the flow remains laminar during the entire combustion process (laminar finite-rate model); (ii) the multi-step chemical kinetics in the gas-phase (volumetric) and complete (or deep) reaction models for catalytic surface reactions occur with rate constants that exhibit Arrhenius-type dependence (Equation (6)); and (iii) explosives of constant concentrations are present in the control volume of the mixture with air. In Fluent^®^, the concentrations of the reactants need to be specified on the basis of mass fractions.

The volumetric Joule heat generated by an electric current through a resistive heating element can be calculated from Ohm’s law as follows:

(8) $$Q=I^{2}R=I^{2}\rho \frac{l}{A}\to q=\frac{Q}{V}={\left(\frac{I}{A}\right)}^{2}\rho$$

Fluent^®^ does not provide a solution for Joule heating, so a user-defined function (UDF) code was implemented in the Fluent^®^ case file. In this UDF code, the electrical conductivity values were defined as the diffusivity of the solid phase potential in the solid zones. Thermal analyses of catalytic oxidation based on species transport and the gas phase coupled with the surface oxidation models were then performed.

Numerical simulations were performed using a three-dimensional (3-D) model, assuming steady-state conditions for laminar flow, coupled with the species transport equations in the gas phase, along with surface reaction. Hexagonal and gradient meshing techniques were used to discretize the equations. A solid model was generated using the Gambit^®^ software for use in the thermal analysis performed using Fluent^®^. The size of the simulation volume was determined by estimating the thickness of the thermal boundary layer due to natural convection over the heated surface, using the following equation [24]:

(9) $$\delta_{T}=0.0014Ra_{\delta }^{0.24}$$

where *Ra_δ_* is the Rayleigh number. For the temperature at the maximum actuation current (e.g., 20 mA), the thickness of the boundary layer is approximately 200 µm. The convective heat transfer coefficient (*h*) was calculated from the value of the Nusselt number obtained from the pure conduction correlation (Equation (10)) and was observed to be consistent with the value obtained from experimental data (*h* = 700 W/m^2^-K) [25]:

(10) $$Nu=\frac{hL}{k}=1$$

where *L* is the characteristic length (m), estimated by assuming *L* = Volume/Surface Area, and *k* is the thermal conductivity (W/m-K) for the mean temperature *T_m_* = (*T_s_* + *T_∞_*)/*2*.

#### 2.3.2. Finite Element Analysis

The CFD tool provides a powerful and flexible numerical framework for modeling fluid flow and performing associated convection heat transfer calculations, but it does not have built-in advanced solid mechanics analysis capabilities for performing thermo-mechanical stress analysis. On the other hand, the FEA tool provides advanced solid mechanics analysis capabilities. To calculate the mechanical deflection produced by the thermal stress at the surface of a microcantilever, a UDF code for CFD/FEA thermal mapping was implemented in Fluent^®^. The meshing and scaling of the models should be consistent in both Fluent^®^ and Ansys^®^.

### 2.4. Temperature-Dependent Properties

The temperature dependence of the thermo-physical properties (e.g., specific heat capacity, dynamic viscosity, and thermal conductivity) and thermo-chemical properties (e.g., diffusivity) is considered in the numerical models to enhance their numerical accuracy. The values of these properties were obtained from various reports in the literature and from the Fluent^®^ database, which is derived from a National Institute of Standards and Technology (NIST) database [26]. In the 3-D models, the binary diffusion coefficient (Equations (11)–(15)) is calculated using the relationships provided by Reid *et al.* [27]. This theoretical model relies on the values of the hard-sphere collision diameter and the Lennard-Jones energy parameters, which are summarized in Table 4.

**Table 4:** Hard-sphere collision diameter and Lennard–Jones energy parameter for various species.

Species	*σ* (Å)	*ε / k_b_* (K)
H_2_	2.827	59.7
H_2_O	2.641	809.1
N_2_	3.798	71.4
O_2_	3.467	106.7
CO	3.690	91.7
CO_2_	3.941	195.2
C_3_H_6_	4.807	248.9
C_3_H_6_O	4.670	443
C_3_H_8_O	4.937	393.42

(11) $$D_{ij}=\frac{0.0266T^{3/2}}{p{M_{ij}}^{1/2}{\sigma_{ij}}^{2}{\text{Ω}}_{D}}$$

(12) $$M_{ij}=2{\left[{M_{i}}^{-1}+{M_{j}}^{-1}\right]}^{-1}$$

(13) $$\sigma_{ij}=\left(\sigma_{i}+\sigma_{j}\right)/2$$

(14) $${\text{Ω}}_{D}=\frac{1.06036}{{\left(T^{*}\right)}^{0.15610}}+\frac{0.19300}{\exp\left(0.47635T^{*}\right)}+\frac{1.03587}{\exp\left(1.52996T^{*}\right)}+\frac{1.76474}{\exp\left(3.89411T^{*}\right)}$$

(15) $$T^{*}=k_{B}T/{\left(\varepsilon_{i}\varepsilon_{j}\right)}^{1/2}$$

where *σ* is the hard-sphere collision diameter (Å) and *ε/kB* is the Lennard-Jones energy (K). The mixture viscosity and thermal conductivity were determined using the kinetic theory of gases and the ideal gas mixing law, respectively.

## 3. Fabrication Procedure and Experimental Apparatus

### 3.1. Fabrication of the Microcantilever Array

**Figure 3:**
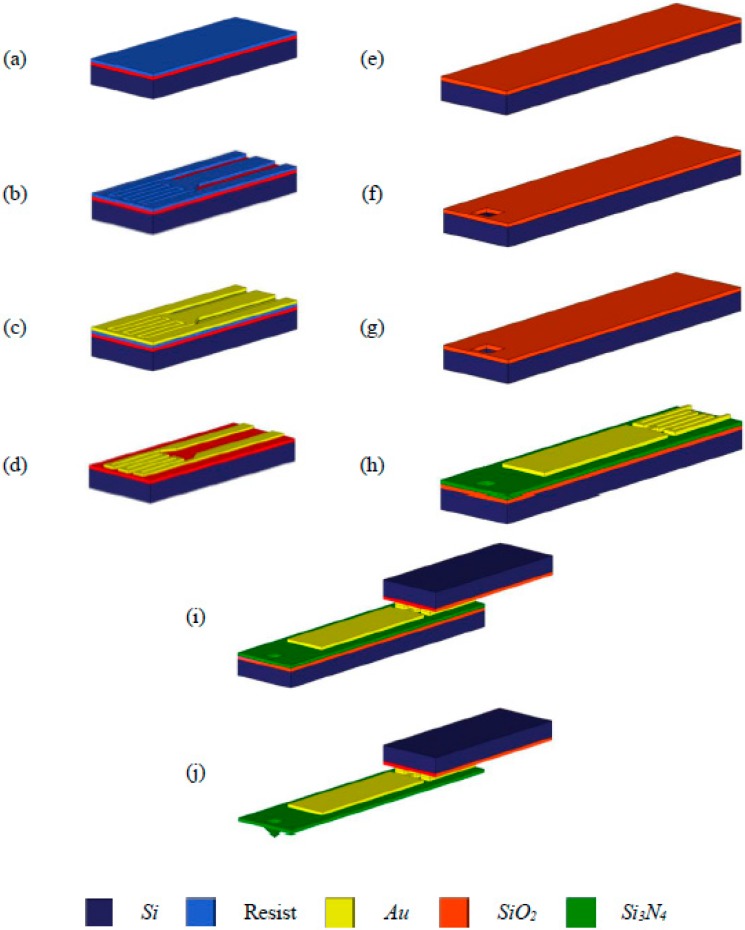
3-D isotropic view of the micro-cantilever fabrication scheme: (**a**) deposition of resist layer on oxidized (SOI) wafer; (**b**) resist layer patterning for interconnect metal; (**c**) Cr/Pt/Au (30/60/1000 nm) layer deposition; (**d**) Au lift-off; (**e**) oxidation of Si (SOI) wafer; (**f**) SiO_2_ patterning; (**g**) anisotropic Si etch; (**h**) Si_3_N_4_ deposition and its patterning, lift-off resist patterning, and Cr/Pt/Au (30/60/400 nm) deposition and lift off; (**i**) Au-Au thermal compression bonding; and (**j**) Si release (TMAH etch: Si mold wafer etch and Si handle etch).

**Figure 4:**
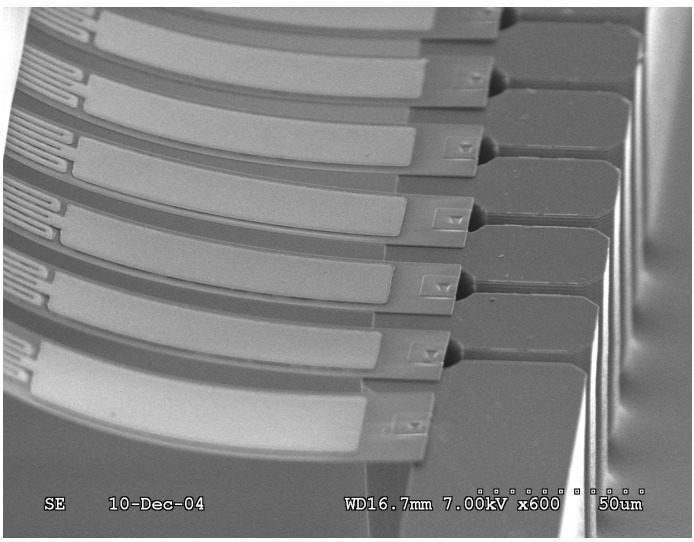
Optical microscopy image of micro-fabricated bimorph microcantilever array integrated with heating element at their base.

The fabrication process of the thermal bimorph microcantilever array (Active Pen^TM^ platform) is illustrated in Figure 3. For the interconnect wafer (Figure 3a–d)), the backside of an oxidized (1 μm) wafer is patterned, and the oxide is etched. After the resist is applied to the front, it is patterned, and a Cr/Pt/Au (30/60/1000 nm) film is deposited by physical vapor deposition (PVD) at low pressure (2 × 10^−7^–7 × 10^−7^ torr) and patterned using the “lift-off” process. For the tip wafer (Figure 3e–h), the oxidized wafer is patterned into small squares and used as a mask in an anisotropic silicon etching process to form a pyramidal tip. After a low-stress and low-stress-gradient Si_3_N_4_ film is deposited on an oxidized silicon wafer a priori by low pressure chemical vapor deposition (LPCVD), it is patterned and dry-etched. The resist is then applied and patterned. This is followed by patterning of the Cr/Pt/Au (30/60/400 nm) thin film by physical vapor deposition and the “lift-off” process (which is similar to the previous metal deposition process). Finally, the two wafers are aligned by infrared (IR) alignment, clamped together using a Suss MA-6 platform, and bonded by thermal compression bonding at a high temperature (e.g., 350 °C) in a Suss SB-6 bonder. The handle and tip wafers were etched in tetramethylammonium-hydroxide (TMAH), and the sharpening oxide layer was removed using buffered oxide etching (BOE). The thermal stresses induced during bonding can exceed the elastic limit and are not completely relieved when the substrates are returned to room temperature. That is, cooling down to room temperature generates residual stresses. An initial curvature is therefore induced in the microcantilevers by the residual stresses that are generated during cooling down to room temperature after thermal bonding. Prior to the experiments, the microcantilevers were cleaned using the plasma cleaning method (with a gas mixture of 20% O_2_ and 80% Ar) using a reactive-ion etching (RIE) instrument. Figure 4 shows the micro-fabricated microcantilever array, which is hooked up to a flexible printed circuit board (PCB) (courtesy of NanoInk, Inc., Campbell, CA, USA). Each cantilever can be actuated individually by a separate electrical connection.

### 3.2. Experimental Apparatus and Procedure

**Figure 5:**
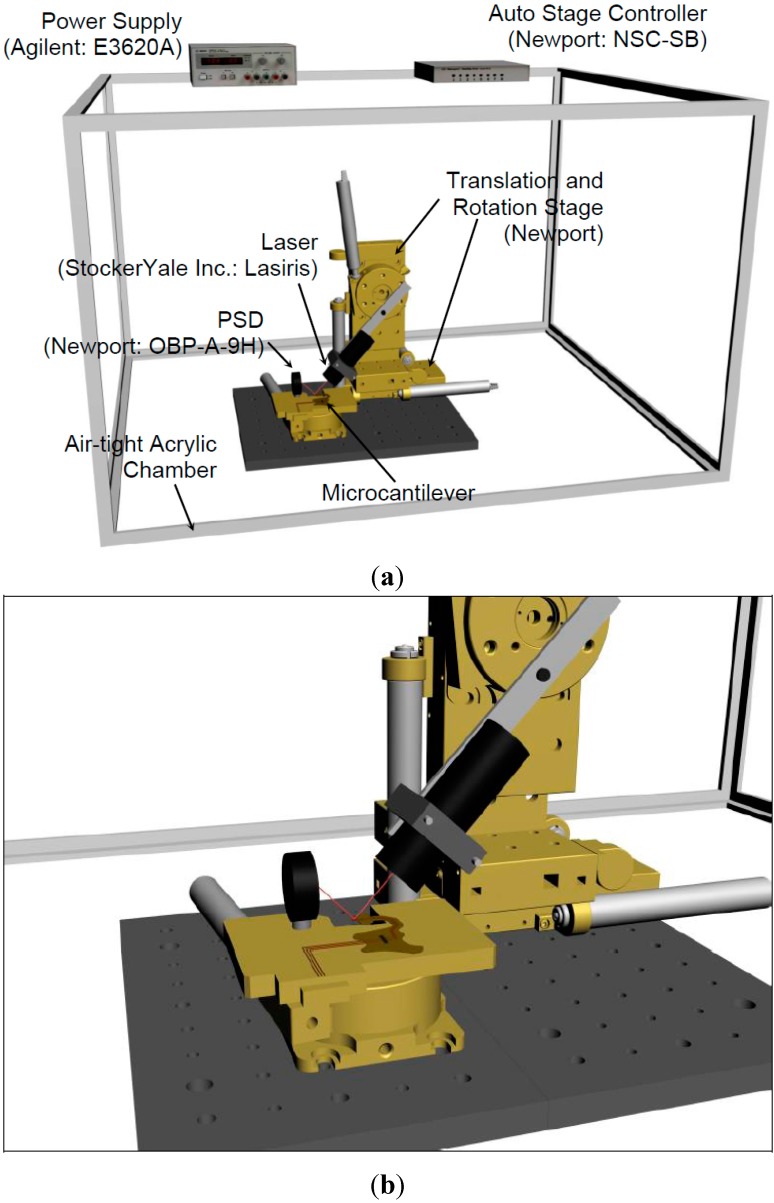
Schematic of the experimental apparatus based on the optical-lever method used in this study for explosive detection: (**a**) experimental setup for detection; and (**b**) sensing of reflected light using PSD.

Nano-scale deflections are commonly monitored using the “optical detection (lever) method,” illustrated in Figure 5. This technology was developed in 1988 for use in atomic force microscopy (AFM) [28]. The experimental setup consists of an airtight acrylic chamber (an environmental control chamber), a platform to support and control the movement of the laser source, a platform for the microcantilever array, and a position-sensitive detector (PSD) to detect the laser beam spot reflected from the surface of each microcantilever. The experimental apparatus was placed inside the environmental control chamber, which was constructed from rectangular acrylic walls and a hinged door made from 1/2-in-thick acrylic sheets, as shown in Figure 5. To render the acrylic chamber airtight, silicone was used to seal the edges inside the box, and an aluminum tape insert was used to seal the edges along the exterior of the box. Weather stripping was used as a sealant between the door and the front wall of the chamber. Inside the chamber, a low-power laser (1 mW, 635 nm) was affixed to a semi-automated stage with four axes of motion (assembled from Newport components). The Newport stage system (Figure 5) supports the laser and can be actuated remotely for laser beam alignment with the cantilever axes in the nano-calorimeter apparatus. To alter the position of the laser, a remote-controlled stage can also be used to rotate the cantilever array. Hence, laser alignment and cantilever positioning can be accomplished without perturbing the environmental chamber.

Each experiment was performed in two separate steps. First, a control (baseline) experiment was performed under ambient atmospheric conditions, and second, an experiment was performed in the presence of explosive vapors at the equilibrium vapor concentration. The bending response of the microcantilever as a function of the actuation current in the uncontaminated air environment was compared to that in air saturated with the vapor of the explosive materials (or VOCs) chosen for use in this study. As shown in Figure 5, the laser beam incident on the microcantilever surface is reflected by the gold coating onto the PSD. The actuation current (for heating the micro-heaters and therefore for actuating the microcantilever beam) was increased from 0 to 20 mA at 2 mA intervals. The resulting deflection of the microcantilever beam was tracked by measuring the voltage values corresponding to the position of the reflected laser beam obtained from the PSD. Once the data were collected for the control experiment, the explosive sample was placed in a small bowl that was placed inside the chamber. The sample was placed in the environmental control chamber for approximately thirty minutes to ensure saturation conditions for the vapor emanating from the explosive sample. The results were then recorded and compared to the results obtained from the control experiments.

### 3.3. Uncertainty

The change in the deflection angle of the microcantilevers was measured as a function of the actuation current. The total experimental error in the angle measurement is given by [10]:

(16) $$\frac{\delta \theta }{\theta }=\sqrt{{\left(\frac{\delta z}{z}\right)}^{2}+{\left(\frac{\delta L}{L}\right)}^{2}}$$

where *θ* is the deflection angle (°), *z* is the change in height (m) from the reference point on the PSD, *L* is the distance (m) from the cantilevers to the PSD, and *δθ* is the error in the calculation of the deflection angle. In this study, the experimental error in measuring the change in angular deflection was found to be less than 0.5%.

## 4. Results and Discussion

### 4.1. Baseline Simulation

**Figure 6:**
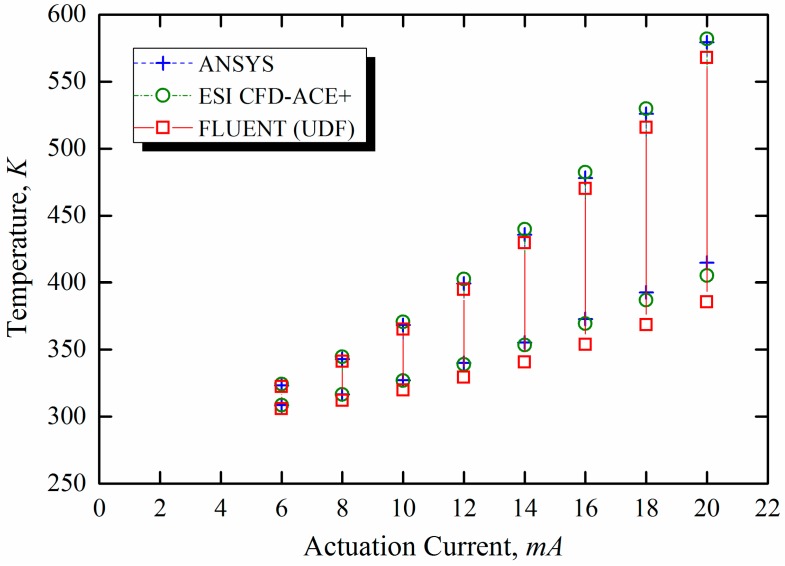
Numerically estimated temperature range of microcantilever heated in air.

Oxidation of VOCs with air was numerically studied at specific initial concentrations in a finite control volume. The chemical kinetics, expressed in Arrhenius form according to Equation (6), were used to model the temperature dependence of the reaction rate and the activation energy for oxidation. The higher surface area-to-volume ratio at the nano-scale was expected to expedite the kinetics of the area-limited catalytic reactions, which means that the chemical reactions were expected to occur only on the catalyst surface provided by the gold coatings on the microcantilevers [15]. The catalytic reaction on the surface of the microcantilevers depends on the core temperature of the heating element. Figure 6 shows the surface temperature range of the electrically preheated microcantilevers in air. To verify the UDF code, the temperature profile calculated using the UDF for ohmic heating in Fluent^®^ was compared with the results of electro-thermo-multiphysics analysis in Ansys^®^ and ESI CFD-ACE+^®^. The numerical results obtained using Fluent^®^, Ansys^®^, and ESI CFD-ACE+^®^ indicate almost the same temperature distributions over the cantilever surface.

### 4.2. Bending Response to Explosive Vapors

In the kinetic-controlled region, the concentration of explosives over the surface is obtained as the general solution to the diffusion equation given by Equation (5).

(17) $$C_{i}(x)=C_{1}e^{mx}+C_{2}e^{-mx}$$

where

$$m={\left(k_{v}/D_{i,air}\right)}^{1/2},\;C_{1}=\frac{C_{i,0}\left(m-k_{s}/D_{i,air}\right)e^{-mL}}{2m\cosh\left(mL\right)+\left(2k_{s}/D_{i,air}\right)\sinh\left(mL\right)},\text{ and }C_{2}=C_{i,0}-C_{1}$$

Prior to the 3-D simulations conducted using the multi-step reaction model and the temperature-dependent material properties, a simple 1-D calculation was performed to verify the theoretical model. The global one-step oxidation model for propane (C_3_H_8_) was used in this calculation. The equation given by Hayes *et al.* [15] for the chemical kinetics is as follows:

(18) $$k_{v}=5.0\times 10^{9}\exp\left(\frac{8.98\times 10^{7}}{T}\right)\;\left({\text{kmol/m}}^{3}\text{-s}\right)$$

(19) $$k_{s}=2.4\times 10^{5}\exp\left(\frac{8.98\times 10^{7}}{T}\right)\;\left({\text{kmol/m}}^{3}\text{-s}\right)$$

**Figure 7:**
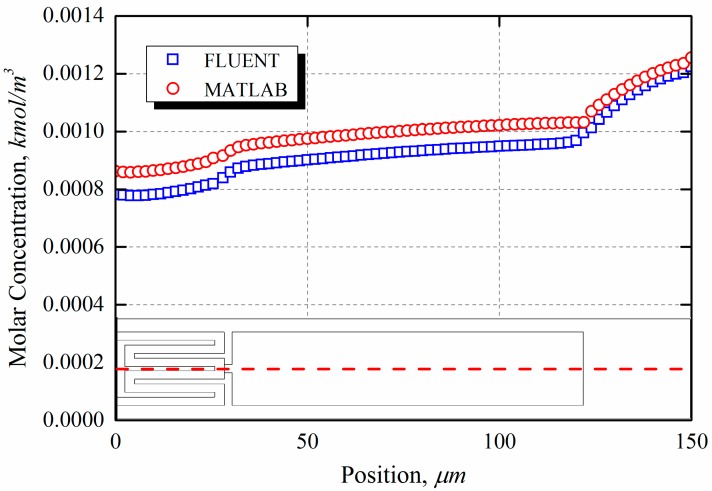
Concentration profiles over the microcantilever surface in the longitudinal direction.

A comparison of the concentration profiles over the heated surface obtained from the 1-D analysis and the CFD simulation is shown in Figure 7. In the 1-D analysis, the concentrations of the explosive vapor varied only with the height. Nevertheless, the predictions obtained from the 1-D analytical model were found to be in good agreement with the results of the CFD simulations.

Complete oxidation of VOCs proceeds with the formation of the oxidation products carbon dioxide (CO_2_) and water vapor (H_2_O), as summarized in Table 1. The species of the explosive vapor are assumed to be uniformly distributed throughout the control volume. The constant mole (or mass) fraction for the gas phase corresponds to the vapor–liquid equilibrium (VLE) mole fraction calculated from Dalton’s law of partial pressures for each species at room temperature (293.15 K). The temperature-dependent vapor pressure (*P_i_^sat^*) of the VOCs is defined by the Antoine equation (Equation (20)) [29]:

(20) $$\log{P_{i}}^{sat}=A-\frac{B}{T+C}$$

where *A*, *B*, and *C* are the Antoine coefficients. The initial conditions for concentration and the enthalpy for phase change are listed in Table 1.

**Figure 8:**
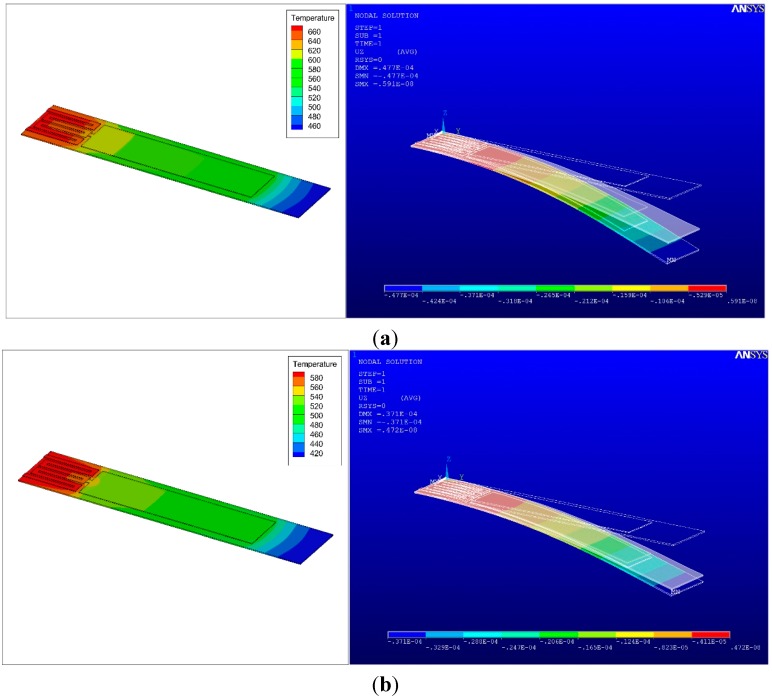
Surface temperature profile by nano-scale combustion reactions on the surface of the microcantilevers and bending response caused by bimetallic effect at 20 mA (**a**) acetone and (**b**) 2-propanol (note: translucent images represent the deflection in air).

**Figure 9:**
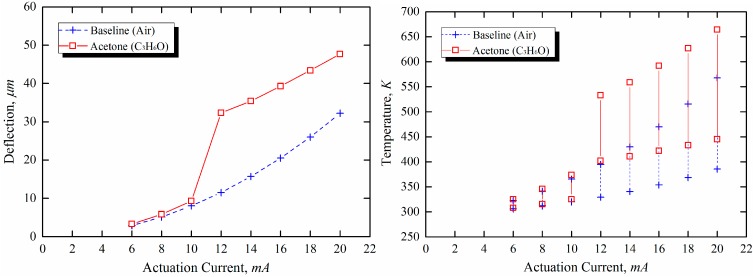

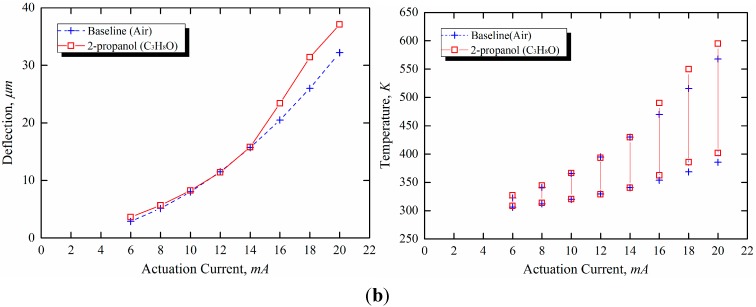
Simulation of the resultant deflection and temperature changes due to nano-scale combustion as a function of actuation current (from 6 mA to 20 mA at 2mA intervals) (**a**) acetone and (**b**) 2-proapnol.

The oxidation reactions of explosives are highly exothermic; hence, the surface temperature is increased by the additional thermal energy generated by the catalytic combustion reactions (*i.e.*, no-flame processes) of VOC vapors on the thin film of gold, as shown in Figure 8 and Figure 9. Accordingly, a downward bending response of the microcantilevers is caused by the thermal bimorph effect, as shown in Figure 8. Figure 9 shows the resultant deflection and temperature distribution of the microcantilever as a function of the actuation current. The amount of gas consumed during the oxidation reactions is proportional to the concentration of the explosive vapor around the cantilever. The difference in the heat of combustion between acetone and 2-propanol is not significant. However, because the vapor pressure of acetone is higher than that of 2-propanol, the surface temperature enhancement due to combustion reactions is augmented in the case of acetone.

Larger deflections are achieved by design optimization, but the noise signal produced by flow or thermally induced excitations can reduce the deflection sensitivity [30]. Thus, a higher resonant frequency is required to improve the signal-to-noise ratio of the deflection measurements. The sensitivity relationship aforementioned provides a guideline for the geometric design of the microcantilever-based sensor. On the other hand, for the same geometrical configuration, the sensitivity can be enhanced by increasing the change in surface stress, which is proportional to the temperature changes produced by the reaction stimulus of the explosive vapors. That is, the sensitivity of the deflection measurements is experimentally evaluated as the ratio of the input values to the output results (*i.e.*, the change in the deflection in relation to the electrical actuation) [31]. Equation (21) expresses the change in sensitivity for an explosive vapor as a function of the actuation current.

(21) $$Sensitivity(S)=\frac{outputchange}{load}=\frac{\Delta z/z}{I}$$

**Figure 10:**
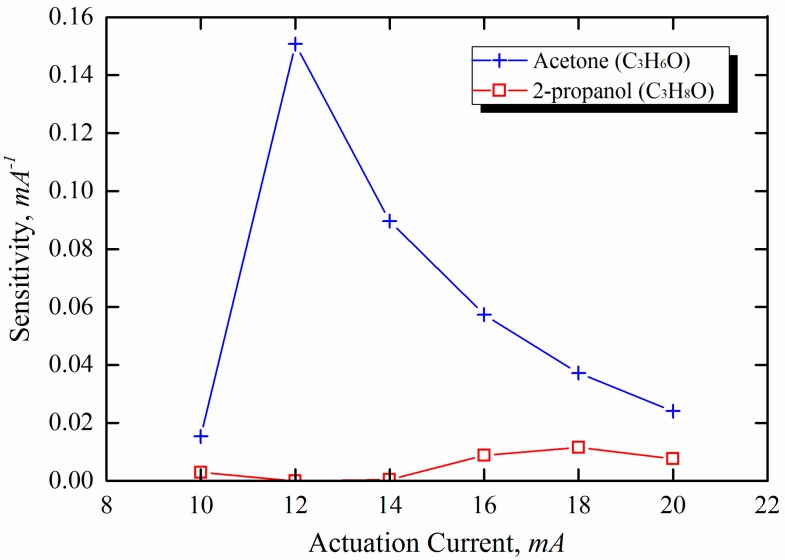
Actuation current dependence of sensitivity for microcantilever-based sensor.

Based on the results shown in Figure 10, the optimal operating current can be defined as the current value that yields the highest sensitivity. The highest sensitivities of acetone and 2-propanel were found to occur in different actuation current regions.

**Figure 11:**
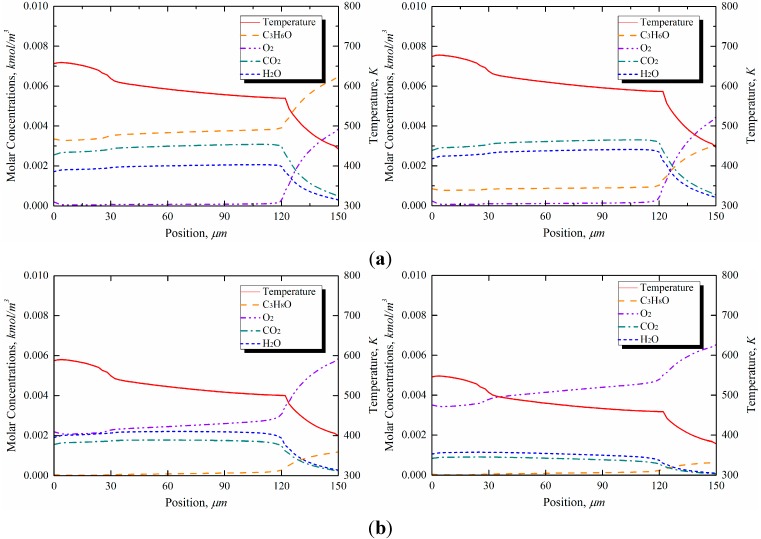
Surface coverage of species and wall temperature profile in different mole fractions (or concentrations) (**a**) acetone (explosive:air = (**Left**) 0.243:0.598 and (**Right**) 0.122:0.694); and (**b**) 2-propanol (explosive:Air = (**Left**) 0.044:0.756 and (**Right**) 0.022:0.773).

Figure 11 shows the concentration profiles of the complete oxidation and the intermediate oxidation products produced during the catalytic oxidation of acetone and 2-propanol over the microcantilevers, for an actuation current of 20 mA. Based on the simulation results, the maximum surface temperature at the applied actuation current was found to be 572 K at 20 mA, as shown in Figure 9, which is below the auto-ignition temperatures of 738 K for acetone and 672 K for 2-propanol. Oxidation that occurs below the auto-ignition temperature yields both complete oxidation products, such as CO_2_ and H_2_O, and intermediate products, such as CO.

The effect of sensor performance on the variation in concentration was explored numerically in this study. Because of the high vapor pressure of acetone, the mole fraction of acetone in a binary gas mixture is much higher than that of 2-propanol, as shown in Table 1. A mixture of air and acetone can therefore be regarded as a richer mixture (lower air-fuel ratio, AFR). Hence, this reaction, being diffusion-limited, is controlled by the oxygen concentration. As shown on the right-hand side of Figure 11, by reducing the concentration of acetone, the mixture yields a higher AFR, and the temperature is increased. However, because a mixture of air and 2-propanol is lean, the surface temperature is also decreased when the concentration of gas is reduced by a factor of two.

### 4.3. Experimental Results

**Figure 12:**
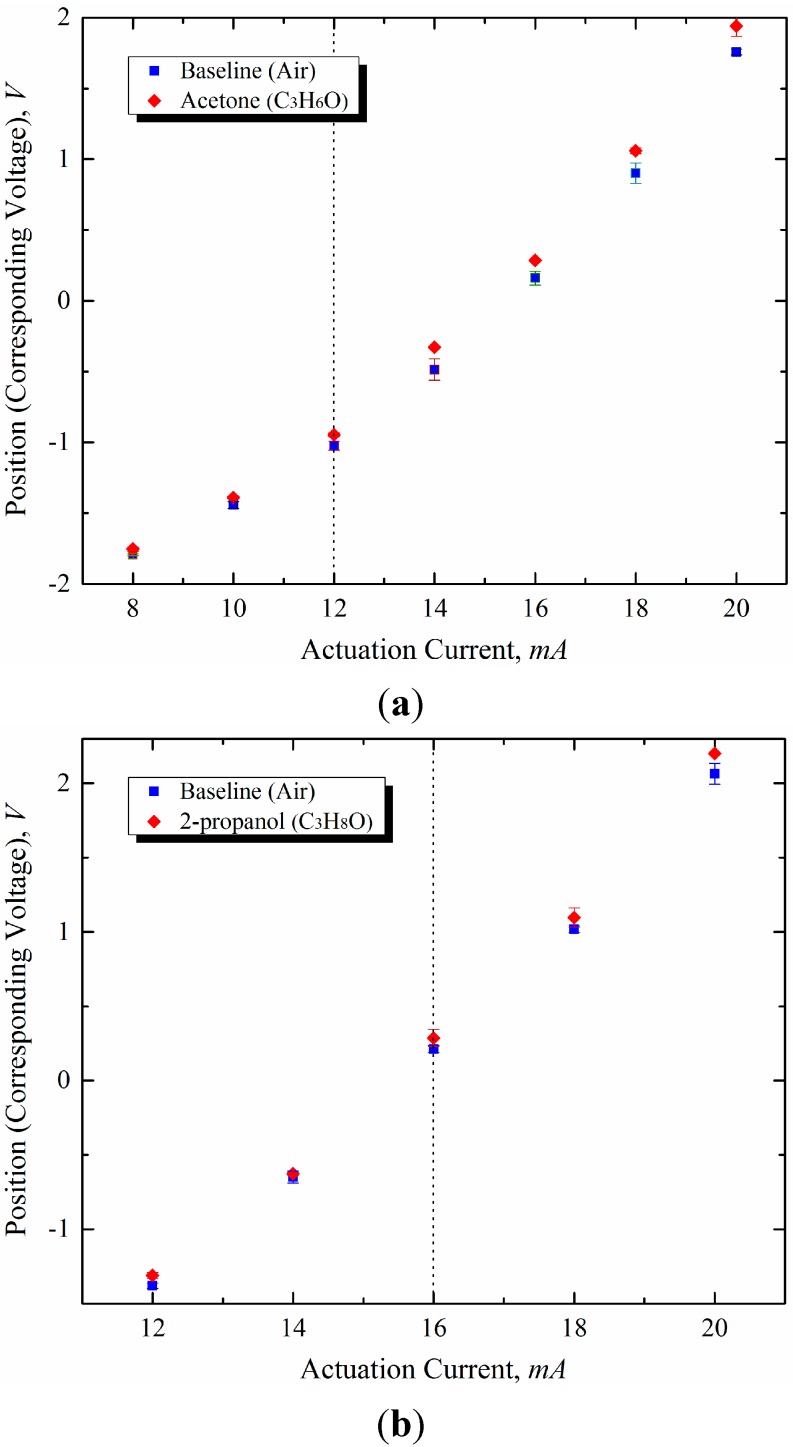
Experimental results of microcantilever deflection based on optical lever method in explosive sensing (**a**) acetone and (**b**) 2-propanol.

The numerical predictions were validated experimentally using VOCs (e.g., acetone and 2-propanol). The experimental results are plotted in Figure 12. The values plotted were obtained by averaging three measurements for each actuation current value. The trends observed in the results show that the change in deflection differs from that in the control experiments (performed in air) at a specific value of the actuation current. For both acetone and 2-propanol, the trends observed in the experiments were consistent with the trends predicted by the numerical models. The value of the actuation current at which the change in deflection deviates from that in the control experiments is called the threshold current. The threshold currents were approximately 12 mA for acetone and 16 mA for 2-propanol, respectively. However, the small deviation due to the conversion from 2-propanol to acetone at a low temperature, present in the simulation results (*i.e.*, Figure 9) was not clearly shown because of the noise of the measured signal.

As mentioned previously, the deflection sensitivity of acetone and 2-propanol is characterized by the threshold currents for activation of catalytic oxidation over the microcantilever surface. As shown in Figure 10, acetone exhibits a higher sensitivity in the lower actuation current region than 2-propanol. On the other hand, the high sensitivity of 2-propanol was demonstrated at currents higher than the threshold current of acetone. Therefore, the selectivity in the detection of different explosive vapors can be enhanced by adjusting the actuation current based on the high-sensitivity region. In reality, the sensitivity depends on the actual concentrations, the ambient temperature, the humidity, and the mixture ratio (*i.e.*, whether the mixture is lean or rich). In conclusion, it was observed that the deflection characteristics evident from the experimental data were consistent with the results predicted from the numerical simulations.

## 5. Summary and Conclusions

In this study, the static response of microcantilevers in the presence of air and explosive vapors was characterized experimentally and by performing numerical simulations. To explore the bending response of the microcantilevers, the change in deflection caused by the bimetallic effect was measured experimentally. Numerical analyses based on an electro-thermo-mechanical coupling model were performed using a UDF in Fluent^®^ and Ansys^®^. In the presence of different energetic materials, the microcantilevers exhibited different trends in their bending response with increasing actuation current, both in the numerical simulations and the experimental investigations. Hence, this detection scheme can be used to identify various explosives according to the unique bending response signature displayed by a thermal bimorph microcantilever sensor as a function of the actuation current. In addition, the results show that the thermal conductivity of the microcantilever material can affect the sensitivity of the device performance. Hence, the sensor sensitivity can be enhanced using coatings made of high-thermal-conductivity materials (e.g., carbon nanotubes (CNTs)) on the microcantilever surfaces (e.g., using dip-pen nanolithography (DPN)). The approach explored in this study can be implemented in a portable detection platform or integrated instrument for remote monitoring and real-time detection of explosives.
